# Item response theory-based measure of global disability in multiple sclerosis derived from the Performance Scales and related items

**DOI:** 10.1186/s12883-014-0192-1

**Published:** 2014-10-03

**Authors:** Eric Chamot, Ilya Kister, Gary R Cutter

**Affiliations:** Department of Epidemiology, University of Alabama at Birmingham School of Public Health, 1665 University Blvd. Suite 217H, Birmingham, AL 35294-0022 USA; New York University-Multiple Sclerosis Care Center, Department of Neurology, New York University School of Medicine, 240 East 38th Street, New York, NY 10016 USA; Department of Biostatistics, University of Alabama at Birmingham School of Public Health, 1665 University Blvd. Suite 410B, Birmingham, AL 35294-0022 USA

**Keywords:** Multiple sclerosis, Disability evaluation, Patient-reported outcome measure, North American Research Committee on Multiple Sclerosis (NARCOMS) registry, Factor analysis, Bifactor model, Item response theory

## Abstract

**Background:**

The eight Performance Scales and three assimilated scales (PS) used in North American Research Committee on Multiple Sclerosis (NARCOMS) registry surveys cover a broad range of neurologic domains commonly affected by multiple sclerosis (mobility, hand function, vision, fatigue, cognition, bladder/bowel, sensory, spasticity, pain, depression, and tremor/coordination). Each scale consists of a single 6-to-7-point Likert item with response categories ranging from “normal” to “total disability”. Relatively little is known about the performances of the summary index of disability derived from these scales (the Performance Scales Sum or PSS). In this study, we demonstrate the value of a combination of classical and modern methods recently proposed by the Patient-Reported Outcome Measurement Information System (PROMIS) network to evaluate the psychometric properties of the PSS and derive an improved measure of global disability from the PS.

**Methods:**

The study sample included 7,851adults with MS who completed a NARCOMS intake questionnaire between 2003 and 2011. Factor analysis, bifactor modeling, and item response theory (IRT) analysis were used to evaluate the dimension(s) of disability underlying the PS; calibrate the 11 scales; and generate three alternative summary scores of global disability corresponding to different model assumptions and practical priorities. The construct validity of the three scores was compared by examining the magnitude of their associations with participant’s background characteristics, including unemployment.

**Results:**

We derived structurally valid measures of global disability from the PS through the proposed methodology that were superior to the PSS. The measure most applicable to clinical practice gives similar weight to physical and mental disability. Overall reliability of the new measure is acceptable for individual comparisons (0.87). Higher scores of global disability were significantly associated with older age at assessment, longer disease duration, male gender, Native-American ethnicity, not receiving disease modifying therapy, unemployment, and higher scores on the Patient Determined Disease Steps (PDDS).

**Conclusion:**

Promising, interpretable and easily-obtainable IRT scores of global disability were generated from the PS by using a sequence of traditional and modern psychometric methods based on PROMIS recommendations. Our analyses shed new light on the construct of global disability in MS.

**Electronic supplementary material:**

The online version of this article (doi:10.1186/s12883-014-0192-1) contains supplementary material, which is available to authorized users.

## Background

There is an acute need for a reliable and valid quantitative outcome measure of “global disability” in multiple sclerosis (MS) from the patient’s perspective. The North American Research Committee on Multiple Sclerosis (NARCOMS) registry, a volunteer registry that represents approximately 10% of the U.S. MS population affords a unique opportunity to develop and validate such a measure. Since 1998, the NARCOMS registry has employed the *Performances Scales* (PS) to assess perceived disability in adults living with MS [1]. Single-item PS were originally developed for eight domains of function (mobility, hand function, vision, fatigue, cognition, bladder/bowel, sensory, and spasticity) [1]. To increase content validity [2,3], three more measures were added in 2001 to assess disability associated with pain [4], depression [5], and tremor/coordination [6]. Responses are recorded on a 6-point ordinal scale (0 normal, 1 minimal, 2 mild, 3 moderate, 4 severe, and 5 total disability) except for the mobility PS which is scored from 0 to 6.

The measurement properties of each individual PS are generally excellent. Non-response rates are low (1%-5%) and test-retest reliability high (median, 0.82, range 0.65-0.91) [1]. Evidence of criterion and construct validity is strong for virtually all the scales (Additional file 1) [4-9]. Although the PS have generally been analyzed separately, several authors have added raw scores on the eight original PS to form an ordinal summary index of disability, referred to as *Performance Scales Sum* (PSS-8) [1,7,10-12]. The PSS-8, has shown favorable properties in terms of internal consistency (Cronbach’s alpha, 0.78), test-retest reliability (intraclass coefficient of correlation, 0.89), and criterion validity (correlation with EDDS, 0.62-0.64; correlation with MSFC, 0.58) [1,7]. Growing evidence also supports its discriminant and incremental validity [1,10]. Furthermore only minimal response shift has been detected when administering the PS repetitively over a 1-year period [11].

An important unresolved question about the PS is that of whether a single sum score, such as the PSS-8, adequately reflects underlying global disability or whether two or more summary scores are needed to validly capture information on disability domains assessed by the PS. For instance, recent factor analysis of 7 of the original 8 PS (vision scale excluded, PSS-7) suggested that a better representation of a patient’s disability might be obtained with two separate scores─one combining the mobility, spasticity and bladder/bowel PS, and the other combining the hand function, fatigue, sensory, and cognition PS [11].

We and others have described how categorical factor analysis and bifactor analysis could help uncover the fine structure of disability in MS [13,14]. In this article, we apply these methods and related techniques put forward by the Patient-Reported Outcome Measurement Information System (PROMIS) network [15] to the evaluation of the measurement structure of the 11 PS in a large cross-sectional sample of NARCOMS registrants. We explain how results from these analyses informed the Item Response Theory (IRT) calibration of the PS on a single scale of self-assessed global disability. Finally we compare the construct validity of three summary scores of global disability derived using assumptions and calculation methods of varying practicality and accuracy. To appeal to a wide readership, methodological details are presented in Additional file 2.

## Methods

### Study sample

Study data included NARCOMS recruitment surveys collected in 2003–2011. Analyses were restricted to participants who completed the pain, depression and tremor PS, which were not consistently included in each intake survey, and to patients who indicated whether or not they had a confirmed diagnosis of MS. Disability items consisted of the 11 PS and the Patient Determined Disease Steps (PDDS)─a patient-assessed single-item measure of perceived disability that correlates as high as 0.78 with the Expanded Disability Status Scale (EDSS) [16]. Other variables available included calendar year of survey completion, gender, race/ethnicity, age at first symptoms, disease duration, employment status, whether the respondent was on disease modifying therapy (DMT) at enrollment, and year of MS diagnosis.

The total sample was randomly split into a development sample (exploratory analyses) and a validation sample (main analyses).

The NARCOMS Registry is approved by the Institutional Review Board of the University of Alabama at Birmingham.

### Preliminary analyses

After performing traditional descriptive statistics for the 11 PS, we conducted exploratory factor analysis (EFA) in the development sample to obtain initial information as to whether the PS should be aggregated into one or more than one disability measures (Additional file 2) [17,18]. Then, based on EFA results and the literature [19], we used confirmatory factor analysis (CFA) to test the fit of the most promising models to the data of the validation sample (Additional file 2).

### Item calibration and measurement

Although IRT and CFA models belong to the same family of latent variable models, IRT models provide more detailed information about the functioning of each item. IRT methods also present several advantages for rigorous scale development and score interpretation [20]. We performed iterative IRT analysis to accomplish the following: examine whether respondents reliably distinguished between adjacent PS categories (Additional file 2); calibrate the PS according to the assumptions of two closely-related, and similarly plausible, CFA models; and generate corresponding IRT-based scores of disability (Additional file 2) [21]. To facilitate interpretation, all IRT scores were transformed to have mean 50 and SD 15 so that >99% of scores in the NARCOMS sample would range between 5 and 95.

### Construct validity

Construct validity was assessed using known-group comparisons, that is, analysis was performed to compare the means of IRT scores generated in the previous step across the categories of key patient characteristics including PDDS score, age, gender, race/ethnicity, disease duration, and year of assessment. We also assessed the associations of IRT score estimates with unemployment, controlling for other potential predictors of unemployment. We used Mplus 6.1 for general psychometric analyses, IRTPRO 2.1 for IRT calibration and IRT scale score estimation, and Stata 12.1 for the other analyses.

## Results

Of the 12,563 persons who filled a NARCOMS intake questionnaire between 2003 and 2011, 7,851registrants with self-reported diagnosis of MS completed all 11 PS. Nearly 80% of participants were women; 93% were white, and 53% completed their intake questionnaire in 2007 or later. Mean age was 46 years (SD, 11.1); mean age at diagnosis 39 years (SD, 10), and mean disease duration 15 years (SD, 11.3). Two-third of respondents were on disease-modifying therapy; 51% were unemployed.

Except if stated otherwise, all the results below were obtained from the validation sample after revision of PS response options as described in Additional file 2 (i.e., after a first round of analysis indicated that response options should be reduced from 7 to 6 for the mobility PS and from 6 to 5 for all the other PS except the fatigue PS). PSS-11 scores, (i.e., traditional raw summed scores) ranged from 0 to 43 out of a revised maximum total of 46.

### Preliminary analyses

EFA suggested that one or two factors (i.e., underlying dimensions of disability) might satisfactorily explain covariations among PS (Additional file 2) [22]. As a follow-up, we fitted three CFA models to the data (Additional file 2): (1) a *unidimensional model*, where the 11 PS represented a single construct of global disability; (2) a *two-dimensional model* composed of two correlated factors that we loosely referred to as “physical disability” (mobility, hand, bladder/bowel, spasticity and tremor PS) and “mental disability” (cognition, fatigue, pain, sensory, depression, vision PS); and (3) a hybrid *bifactor model* [18,23], where the variability common to all 11 PS was captured by a general factor of global disability, and residual fractions of PS variability not accounted for by the general factor were captured by two auxiliary factors of “physical” and “mental” disability. In this latter model, a strong general factor and weak auxiliary factors would suggest that the structure of the data is “almost” unidimensional, and therefore that Model 1 might be preferred over Model 2 (we refer the readers to our article [14] for a general discussion of Models 1–3).

**Figure 1:**
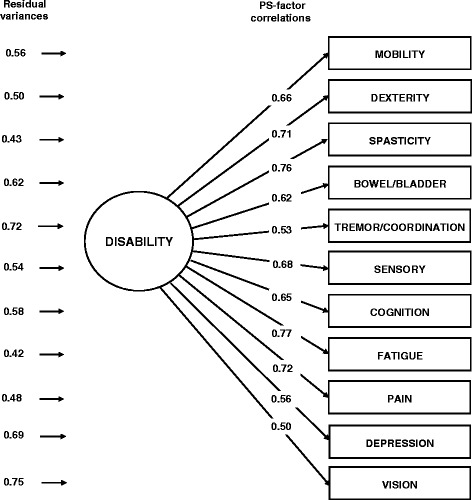
**Unidimensional CFA model of self-assessed neurological disability in NARCOMS registrants.** Note: “Disability” represents a latent factor, i.e., a not directly observable continuous variable whose scale is inferred from the variability and correlations among PS. “PS-factor correlations” are estimates of the correlations between PS and factor scores. “Residual variances” represent the fractions of PS score variability that are not explained by the factor.

The *unidimensional model* had a mediocre fit, but the PS-factor correlations were all moderate to large (mean, 0.65; range, 0.50-0.77; Figure 1).

The fit of the *two-dimensional* model was only marginally better than that of the unidimensional model. Individual PS correlated 0.52-to-0.80 with their respective factor (means, 0.69 for the physical disability factor and 0.67 for the mental disability factor). Correlation between the two factors was high (0.83). Misfit was primarily due to the sensory PS substantially contributing to both the physical factor and the mental factor.

**Figure 2:**
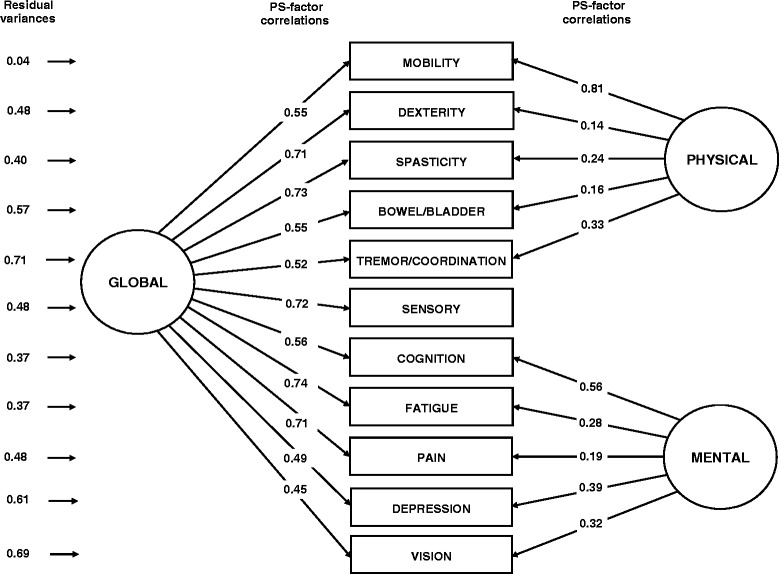
**Bifactor CFA model of self-assessed neurological disability in NARCOMS registrants.** Note: “Global” represents the general factor of global disability; “Physical” and “Mental” represent the auxiliary factors of “physical” and “mental” disability. Correlations among the three factors are all forced to be zero. Thus, the physical and mental factors each explain a fraction of the variability in PS scores left unexplained by the general factor. Comparisons of “Residual variances” in Figure 2 and Figure 1, provide information about the fraction of variability in PS scores that the two auxiliary factors explain above and beyond the general factor.

The bifactor CFA model was specified so that the sensory PS contributed only to the general factor (i.e., to what the physical and mental PS measured in common; Figure 2). The fit of this model was excellent. Correlations between the PS and the factor of global disability were very similar to their counterpart in the unidimensional model. The largest differences in factor-PS correlations were observed for the mobility PS (correlation of 0.55 in the bifactor model vs. 0.66 in the unidimensional model) and the cognition PS (correlation of 0.56 in the bifactor model vs. 0.65 in the unidimensional model) . Both differences matched the accepted standard of ≤0.15 for a small difference [24]. This suggested that the mobility and cognitive PS would be only slightly overrepresented in scores of global disability obtained from the parsimonious, unidimensional, IRT model compared to scores of global disability obtained from the more complex bifactor IRT model. Furthermore, the variance of PS sum scores was decomposed into a large fraction explained by the factor of global disability (79%), a small fraction explained by the two auxiliary factors (11%), and a small fraction of residual error (10%) [25]. Expressed differently, 87.8% of reliable variance in the sum score represented global disability as opposed to domain-specific disability. This result was in line with the finding that only two PS had salient correlations with the factor of residual physical disability (mobility, 0.81 and tremor/coordination, 0.33) and three with the factor of residual mental disability (cognition, 0.56; depression, 0.39; and vision, 0.32). Since the auxiliary factors of a bifactor model are considered to be minor and ill-defined if they include less than three items with item-factor correlations ≥ 0.40-0.50 [26], we concluded that scores of residual physical and mental disability might not be estimated with sufficient accuracy to be of practical importance in less than very large studies.

### IRT calibration and measurement

We fitted both a unidimensional- and a bifactor IRT model to the data. The bifactor IRT model replicated the measurement structure of the bifactor CFA model.

**Figure 3:**
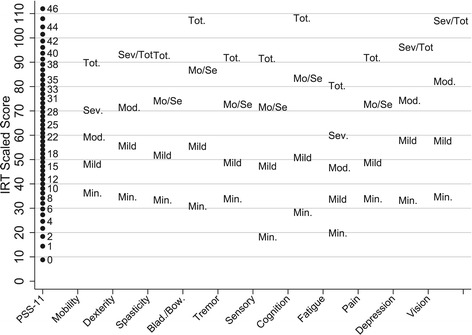
**Relation among Performance Scale (PS) categories, Performance Scales Sum (PSS-11) scores and PS IRT scores of global disability (from unidimensional model) in a sample of NARCOMS registrants.**

The fit of both models was acceptable (Additional file 2), but neither supported the validity of a raw summed score such as the PSS-11 [27]. IRT models indicated in particular that the level of disability corresponding to a given PSS-11 summed score varied as a function of the pattern of responses to PS items. This is illustrated in Figure 3 which describes the relations between IRT scale of global disability from the unidimensional model, raw PSS-11 scores, and standing of PS categories on the IRT scale. The figure, for instance, indicates that a minimum level of fatigue disability contributed less to global disability on the IRT scale than a minimum level of mobility disability. The figure also shows that the distance between two consecutive raw PSS-11 scores varied along the IRT scale continuum. In this situation IRT modeling offered two options. The simplest, but more approximate option was to directly convert raw PSS-11 scores into *IRT summed scores* by aligning the former on the more linear IRT scale. The more rigorous, but less practical option was to estimate *IRT pattern scores* that account for the fact that combinations of PS responses corresponding to distinct true levels of disability may yield the same raw summed score [28,29]. For each PSS raw summed score, one IRT summed score would be generated versus several IRT pattern scores. IRT summed scores would maintain the simplicity of PSS-11 scores, but at the cost of some loss of accuracy. Pattern scores would be more accurate, but too cumbersome to be calculated without a computer application.

To examine the trade-offs among the most promising alternatives, we calculated IRT summed scores and IRT pattern scores of global disability from the unidimensional model, and IRT pattern scores of global disability, residual physical disability, and residual mental disability from the bifactor model. Unless stated otherwise, in what follows “summed scores” and “pattern scores” will refer to IRT scores generated from the unidimensional model, and “bifactor scores” to IRT scores generated from the bifactor model.

**Figure 4:**
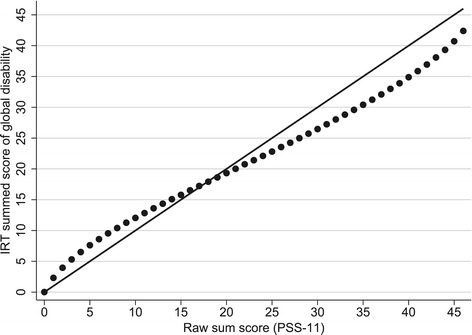
**Relations among the levels of the Performance Scales Sum (PSS-11) score and the IRT summed score of global disability.** Note: To facilitate comparisons, the levels of the IRT summed score were linearly transformed to range from 0 to 46 (i.e., the range of the PSS-11 levels). Thus, had the levels of the two scores been perfectly equivalent, then the dots on the figure would have been aligned on the diagonal line.

A graphical comparison of *PSS-11* summed score levels to corresponding *IRT* summed score levels provided further evidence of the shortcomings of the PSS-11 summed scale─low raw PSS-11 scores (0-to-15) were shown to underestimate corresponding IRT summed scores, while high raw PSS-11 scores (21-to-46) overestimated them. (Figure 4). Therefore, in Additional file 3, we provide a conversion table that appropriately translates raw PSS-11 scores into IRT summed scores of global disability [28,29].

In contrast, differences between IRT *summed* and IRT *pattern* scores of global disability were generally small (mean, −0.07; SD, 2.1) and so were differences between pattern scores and bifactor scores of global disability (mean, 0.0; SD, 2.3). In both cases differences were near zero in the 20-to-80 IRT-score range. These results suggested that the IRT summed score approximation was unlikely to lead to clinically-relevant measurement bias. Overall reliability of the IRT summed scores of global disability was 0.87.

### Construct validity

**Figure 5:**
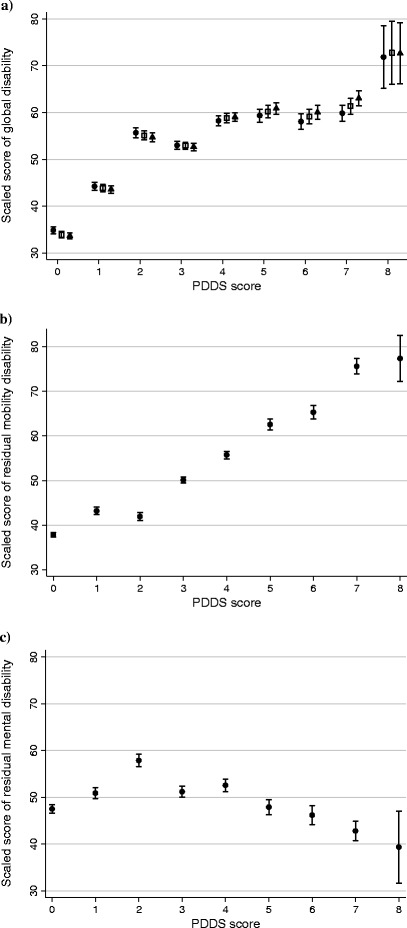
**Associations between Performance Scales-based, IRT-derived, scores of disability and PDDS scores in a sample of NARCOMS registrants. a)** Scores of global disability generated from the bifactor IRT model (●) versus the unidimensional IRT model (□, pattern scores; ▲, summed scores). **b)** Scores of residual physical disability generated from the bifactor IRT model. **c)** Scores of residual mental disability generated from the bifactor IRT model. Note: Although the scores of global disability, residual physical disability, and residual mental disability are all reported as scaled scores (Mean, 50; SD, 15), they are not on the same metric. Error bars represent 95% confidence intervals. Spearman correlations between IRT scores and PDDS scores: Figure 5a) ● 0.60, □ 0.65, ▲0.68; Figure 5b) 0.72; Figure 5c) 0.07.

We observed positive and statistically significant associations between mean IRT scores of global disability and PDDS scores (P < 0.001; Figure 5A). Increases in IRT scores of global disability were sharper over the lower portion of the PDSS scale (0-to-2) than over its higher portion (3-to-8). Differences between summed-, pattern- , and bifactor score estimates of global disability were generally minimal (standardized differences <0.15 except for PDDS 7). Mean bifactor scores of residual physical disability increased significantly and nearly linearly with increasing PDSS scores (P < 0.001; Figure 5B). In contrast, mean bifactor scores of residual mental disability increased significantly over PDSS scores 0-to-2 and then decreased (Figure 5C). The patterns in Figure 5 are consistent with the higher portion of the PDDS scale being biased toward physical disability. Alternatively, these patterns may also indicate that patients experiencing high levels of “mental” disability were less likely to enroll in NARCOMS.

In bivariable analysis, higher IRT scores of global disability were significantly and consistently associated with longer disease duration, older age at assessment, male gender, Native American ethnicity, not receiving DMT, and intake survey completed in 2003–2005 and 2009–2011, compared to 2006–2008 (Table 1). Differences according to the type of score estimate (summed, pattern, bifactor) were small compared to the widths of the confidence intervals for the mean estimates. The pattern of associations between personal characteristics and bifactor scores of residual physical disability (respectively residual mental disability) closely paralleled that between personal characteristics and IRT scores of global disability. This latter result reinforced the hypothesis that the 11 PS were all indicators of one broad underlying construct of global disability.

**Table 1:** **Performance Scales-based, IRT-derived, scaled scores of disability by patient characteristics**

**Characteristic**	**N (%)**	**Global disability**			**Residual physical disability**	**Residual mental disability**
		**Bifactor**	**Unidimensional**	**Unidimensional**	**Bifactor**	**Bifactor**
		**Pattern** ^**a**^ **(95% CI)**	**Pattern** ^**a**^ **(95% CI)**	**Summed** ^**b**^ **(95% CI)**	**Pattern** ^**a**^ **(95% CI)**	**Pattern** ^**a**^ **(95% CI)**
Age at assessment (y.)						
≤ 35	749 (19.1)	45.2 (44.1, 46.2)***	44.6 (43.6, 45.7)***	44.5 (43.5, 45.6)***	42.5 (41.7, 43.4)***	41.2 (40.2, 42.2)***
36 – 55	1760 (44.8)	50.3 (49.6, 51.0)	50.1 (49.4, 50.8)	50.0. (49.3, 50.7)	47.8 (47.1, 48.4)	47.4 (46.6, 48.2)
≥ 56	1417 (36.1)	52.2 (51.5, 53.0)	52.7 (52.0, 53.5)	52.9 (52.2, 53.7)	56.7 (55.9, 57.5)	57.9. (57.0, 58.8)
Disease duration (y.)						
≤ 10	1713 (43.6)	46.2 (45.5, 46.9)***	45.7 (45.0, 46.5)***	45.5 (44.8, 46.2)***	45.2 (44.5, 45.8)***	44.3 (43.6, 45.0)***
11 – 20	1109 (28.3)	51.7 (50.9, 52.6)	51.8 (51.0, 52.7)	51.9 (51.1, 52.7)	50.6 (49.7, 51.5)	50.7 (49.7, 51.7)
≥ 21	1104 (28.1)	54.2 (53.3, 55.0)	54.8 (54.0, 55.6)	55.1 (54.3, 55.9)	56.9 (56.0, 57.8)	58.2 (57.1, 59.2)
Gender						
Female	3102 (79.4)	49.7 (49.2, 50.2)**	49.6 (49.1, 50.1)***	49.5 (49.0, 50.0)***	48.8 (48.3, 49.3)***	48.5. (47.9, 49.1)***
Male	803 (20.6)	51.3 (50.2 52.4)	51.6 (50.5, 52.7)	51.9 (50.8, 53.0)	54.8 (53.7, 55.9)	55.6 (54.4, 56.9)
Race/Ethnicity						
White	3491 (90.2)	49.8 (49.3, 50.3)***	49.8 (49.3, 50.3)***	49.7 (49.3, 50.2)***	50.0 (49.5, 50.5)*	50.0 (49.4, 50.6)*
African American	132 (3.4)	50.7 (48.2, 53,2)	51.0 (48.6, 53.4)	51.5 (49.0, 54.0)	51.8 (49.0, 54.5)	52.1 (48.9, 55.3)
Hispanic/Latino	120 (3.1)	48.1. (45.2, 51.0)	47.9 (45.0, 50.8)	47.8 (44.8, 50.5)	46.5 (43.8, 49.2)	45.9. (42.7, 49.1)
Native American	76 (2.0)	56.7 (53.3, 60.1)	56.6 (53.2, 59.9)	56.7 (53.3, 60.1)	48.3 (45.6, 51.1)	48.0 (44.8, 51.3)
Other	49 (1.3)	53.4 (48.5, 58.1)	53.1 (48.3, 57.9)	53.3 (48.4, 58.3)	47.3. (43.6, 51.0)	46.8. (42.4, 51.2)
Disease modifying therapy						
Yes	2614 (66.6)	49.2. (48.6, 49.8)***	49.2 (48.6, 49.7)***	49.1 (48.5, 49.7)***	48.8. (48.2, 49.3)***	48.6 (47.0, 49.2)***
No	1312 (33.4)	51.6 (50.8, 52.4)	51.7 (50.8, 52.5)	51.8 (51.0, 52.7)	52.4 (51.5, 53.3)	55.6 (54.4, 56.9)
Year of assessment						
2003-2005	1640 (41.8)	50.0 (49.2, 50.7)**	50.3 (49.5, 51.0)*	50.6 (49.8, 51.3)*	51.1 (50.4, 51.9)***	51.3 (50.4, 52.2)***
2006-2008	957 (24.4)	48.9 (47.9, 49.8)	49.0 (48.0, 49.9)	49.0 (48.1, 50.0)	49.1 (48.1, 50.0)	48.9 (47.7, 50.0)
2009-2011	1329 (33.8)	50.8 (50.0, 51.6)	50.4 (49.6, 51.2)	50.0 (49.2, 50.8)	49.3 (48.5, 50.1)	49.2 (48.3, 50.1)

^a^Pattern stands for Bayesian Expected a Posteriori (EAP) score; as many different score estimates were produced for each raw PSS score as the number of item response combinations yielding the PSS score.

^b^Summed stands for EAP Summed Score; only one summary EAP Summed Score was generated for each raw PSS score.

*P < 0.05; **P < 0.01; ***P < 0.001.

Note. The scores of global disability, residual physical disability, and residual mental disability are all reported as scaled scores (S) with mean 50 and SD 15, but they are not on the same metric. All the scores of global disability are directly comparable, but not the scores of residual physical disability and residual mental disability. For instance, because the SD of the score of global disability is much larger than that of the scores of physical and mental disability, a score of global disability of S = 70 represents much more disability than a score of residual physical, or mental, disability of S = 70.

**Figure 6:**
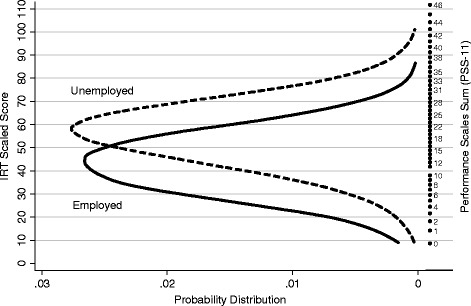
**Relations between distributions of Performance Scales IRT scores (left) and raw Performance Scales Sum (PSS-11) scores (right) among employed and unemployed NARCOMS registrants.** Note: Data on causes of unemployment were not available.

The person-PSS map shown in Figure 6 relates the distributions of IRT summed scores of global disability among employed and unemployed respondents to the estimated standing of raw PSS-11 scores on the IRT scale of global disability. After adjustment for patient characteristics, prevalence of unemployment among respondents independently increased with increasing scores on all three disability measures estimated from the bifactor model (Table 2). Similarly, we also found dose–response relationships between prevalence of unemployment and summed-, and pattern scores of global disability. Pattern scores of global disability were more strongly associated with unemployment than bifactor scores of global disability, presumably because pattern scores of global disability are a form of weighted average of the three bifactor scores of disability (i.e., global-, residual physical-, and residual mental-), and these three scores were all independently associated with unemployment. After controlling for disability scores, prevalence ratio estimates for the personal characteristic variables were remarkably similar across the three regression models, once again suggesting that, despite their imperfections, scores of global disability from the unidimensional model captured most of the disability variance explained by the bifactor model.

**Table 2:** **Associations between IRT-derived disability scores and prevalence of unemployment after adjustment for patient characteristics**

**Characteristic**		**Bifactor model**	**Unidimensional model**	
	**N (%)**	**PR** ^**a**^ **(95% CI)**	**PR** ^**a**^ **(95% CI)**	**PR** ^**a**^ **(95% CI)**
Global disability (scaled score, S)^b^				
S ≤ 33.3	562 (14.3)	1.0^c,d^	1.0^c,d^	1.0^c,e^
33.3 < S ≤ 50	1390 (35.4)	1.50 (1.28, 1.77)	1.83 (1.55, 2.17)	1.73 (1.46, 2.04)
50 < S ≤ 66.7	1428 (36.4)	2.14 (1.83, 2.51)	2.75 (2.34, 3.23)	2.87 (2.46, 3.36)
S > 66.7	546 (13.9)	2.74 (2.33, 3.22)	3.66 (3.12, 4.30)	3.79 (3.24, 4.43)
Residual physical disability (scaled score, S)^b^				
S ≤ 33.3	257 (6.5)	1.0^c,d^		
33.3 < S ≤ 50	1876 (47.8)	1.17 (.99, 1.38)		
50 < S ≤ 66.7	1339 (34.1)	1.56 (1.32, 1.84)		
S > 66.7	454 (11.6)	2.02 (1.70, 2.40)		
Residual mental disability (scaled score, S)^b^				
S ≤ 33.3	236 (6.0)	1.0^c,d^		
33.3 < S ≤ 50	1678 (42.7)	1.11 (.98, 1.26)		
50 < S ≤ 66.7	1783 (45.5)	1.35 (1.19, 1.52)		
S > 66.7	229 (5.8)	1.53 (1.32, 1.78)		
Disease duration (y.)				
≤ 10	1713 (43.6)	1.0^c^	1.0^c^	1.0^c^
11 – 20	1109 (28.3)	1.06 (.98, 1.14)	1.10 (1.02, 1.19)	1.09 (1.01, 1.18)
≥ 21	1104 (28.1)	1.21 (1.13, 1.30)	1.27 (1.18, 1.37)	1.25 (1.16, 1.34)
Age at assessment (y.)				
≤ 35	749 (19.1)	1.0^c^	1.0^c^	1.0^c^
36 – 55	1760 (44.8)	1.01 (.91, 1.11)	1.03 (.93, 1.14)	1.02 (.92, 1.13)
≥ 56	1417 (36.1)	1.22 (1.10, 1.35)	1.31 (1.18, 1.45)	1.28 (1.16, 1.42)
Gender				
Female	3102 (79.4)	1.0^c^	1.0^c^	1.0^c^
Male	803 (20.6)	1.04 (.98, 1.10)	1.08 (1.02, 1.15)	1.08 (1.01, 1.14)
Race/Ethnicity				
White	3491 (90.2)	1.0^c^	1.0^c^	1.0^c^
African American	132 (3.4)	1.14 (.99, 1.31)	1.16 (1.00, 1.33)	1.14 (.99, 1.31)
Hispanic/Latino	120 (3.1)	1.14 (.97, 1.34)	1.11 (.95, 1.31)	1.12 (.94, 1.31)
Native American	76 (2.0)	1.11 (.95, 1.30)	1.10 (.95, 1.28)	1.09 (.94, 1.25)
Other	49 (1.3)	1.23 (1.00, 1.52)	1.24 (1.00, 1.52)	1.16 (.94, 1.43)
Disease modifying therapy				
Yes	2614 (66.6)	1.0^c^	1.0^c^	1.0^c^
No	1312 (33.4)	1.05 (.99, 1.11)	1.08 (1.02, 1.14)	1.06 (1.01, 1.13)
Year of assessment				
2003-2005	1640 (41.8)	1.0^c^	1.0^c^	1.0^c^
2006-2008	957 (24.4)	1.01 (.94, 1.08)	1.01 (.94, 1.09)	1.02 (.95, 1.09)
2009-2011	1329 (33.8)	1.04 (.98, 1.11)	1.01 (.94, 1.07)	1.01 (.95, 1.08)

^a^PR stands for “prevalence ratio”. Because prevalence of unemployment was high, we performed Poisson regression with robust variance estimation instead of logistic regression to obtain unbiased prevalence ratio estimates [30].

^b^IRT scores scaled to have mean 50 and SD 15 in the NARCOMS sample.

^c^Reference category.

^d^Bayesian Expected a posteriori (EAP) estimates of disability (i.e., “IRT pattern scores”).

^e^EAP summed-score estimates of disability (i.e., “IRT summed scores”).

## Discussion

This study supports the notion that information on self-assessed disability elicited by the PS is adequately captured by a single score of global disability. However, several limitations of the standard PSS-11 score were uncovered in this role in terms of excessive number of response categories for 10 items (Additional file 2), underestimation of global disability in the lower part of the scale and overestimation of global disability in the upper part (Figure 4), residual dependency among items from the physical and mental domains of disability (Figure 2), and equal weighing of items (Figure 3). In comparison, the IRT summed score derived from the PSS-11 was shown to be a superior summary measure of patient-assessed global disability in MS with construct validity greater than that of the PSS-11 and similar to that of the harder-to-calculate IRT pattern score.

In this study, we developed a table (Additional file 3) to easily obtain a patient’s IRT summed score of global disability from their raw PSS-11 score. We showed that this IRT estimate has a reliability of measurement (0.87) that is appropriate for both comparisons between groups and individual-level monitoring. To facilitate intuitive interpretation of where a patient stands relative to the distribution of scores in the NARCOMS reference sample, we transformed raw IRT scores into scaled scores with mean 50 and SD 15 in this sample. Finally, we created a diagram that provides graphical information about how each PS contributes to respondents’ perception of their level of overall disability (Figure 3).

We encountered several challenges in data analysis which directed us toward solutions that were less than perfect from a pure measurement perspective. Some experts stress the importance of focusing efforts on well-defined, narrow and strictly unidimensional constructs in order to meet Rasch requirements for fundamental measurement [31,32]. These experts would probably point out that the current PS do not fully cover the breadth of the dimensions of physical disability and, especially, mental disability─they would likely recommend to write and test new PS in order to create two clearly-distinct, and better-defined, unidimensional measures of physical and mental disability. Instead, we adopted the position of those experts who emphasize clinical appropriateness at the cost of small measurement bias [33-36]: i.e., we relied on what the bifactor model enables and gave priority to incorporating in a single summary measure the domains of disability most commonly affected by MS. Analyses suggested that two underlying dimensions of disability could be identified in the data, but that these dimensions were ill-defined and highly correlated. Little reliable information on what we loosely called “physical” and “mental” disability was left in the PS after having extracted information on global disability (this information was provided by the minority of patients affected to markedly different degrees by physical and mental disability). Scores of residual physical disability and residual mental disability were significantly associated with the same patient characteristics. Furthermore, for both scores of residual disability the patterns of associations with patient characteristics was similar to that observed for scores of global disability. With one exception, only the strength of some associations differed slightly depending on the disability score examined. This exception pertained to high PDDS scores, which were positively correlated with high scores of global disability and high scores of residual physical disability, but not with high scores of mental disability. This finding is likely, at least in part to be a reflection of the bias of the upper portion of the PDDS scale toward mobility disability and physical disability in general.

It is crucial for NARCOMS to make full use of the voluminous amount of self-assessed disability data collected during the past decade. For majority of respondents only the 8 original PS are available. One appealing property of IRT is that it will theoretically be possible to use our final IRT model to generate comparable summed and pattern scores of global disability from different subsets of the 11 PS, including the 8 original PS [37]. Future work will be needed to establish how reliable these scores are [35]. Equally important will be the need to determine the longitudinal validity of the IRT scales. Because the IRT framework allows one to model the effects of multiple sources of bias, it will be essential to conduct validation studies not only with IRT score estimates and conventional statistical methods, but also using latent variable methods such as multidimensional IRT modeling and IRT modeling for longitudinal data.

## Conclusion

In summary, our work suggests that ‘global disability’ due to MS is a statistically validated construct that may be readily assessed with the simple and quick-to-administer patient-rated PS. Although, this work was concerned with methodological issues involved in deriving such a score of global disability, it has implications for our understanding of the disease. We showed that self-assessed disability in MS can be conceptualized as a multifaceted construct encompassing elements of both physical and mental disability. Although all the elements considered were closely correlated, the balance between them varied among patients with identical global disability score. This result, derived based on statistical grounds, challenges the traditional view implicit in accepted disability scales (e.g. EDSS), that ‘physical’ disability is of primary import in MS.

## Additional files

Additional file 1:
**Criterion validity of Performance Scale Sum (PSS, raw total score) and individual Performance Scales in cross-sectional studies.**
Additional file 2:
**Methodological details.**
Additional file 3:
**Performance Scales Sum (PSS-11) score to IRT summed score of global disability conversion table.**

## Abbreviations

CI: Confidence interval
CFA: Confirmatory factor analysis
DMT: Disease modifying therapy
EAP: Bayesian expected a posteriori
EDSS: Expanded disability status scale
EFA: Exploratory factor analysis
IRT: Item response theory
MS: Multiple sclerosis
NARCOMS: North American Research Committee on Multiple Sclerosis
PDDS: Patient Determined Disease Steps
PROMIS: Patient-Reported Outcome Measurement Information System
PS: Performance Scales
PSS-7 (8: 11), Performance Scales Sum, 7-, 8-, and 11-item versions respectively
SD: Standard deviation

## References

[1] Schwartz CE, Vollmer T, Lee H (1999). Reliability and validity of two self-report measures of impairment and disability for MS. North American Research Consortium on Multiple Sclerosis Outcomes Study Group. Neurology.

[2] Gruenewald DA, Higginson IJ, Vivat B, Edmonds P, Burman RE (2004). Quality of life measures for the palliative care of people severely affected by multiple sclerosis: a systematic review. Mult Scler.

[3] Mitchell AJ, Benito-León J, González J-MM, Rivera-Navarro J (2005). Quality of life and its assessment in multiple sclerosis: integrating physical and psychological components of wellbeing. Lancet Neurol.

[4] Marrie RA, Cutter G, Tyry T, Hadjimichael O, Vollmer T (2005). Validation of the NARCOMS Registry: pain assessment. Mult Scler.

[5] Marrie RA, Cutter G, Tyry T, Campagnolo D, Vollmer T (2008). Validation of NARCOMS Depression Scale. Int J MS Care.

[6] Marrie RA, Goldman M (2011). Validation of the NARCOMS Registry: Tremor and Coordination Scale. Int J MS Care.

[7] Marrie RA, Goldman M (2007). Validity of performance scales for disability assessment in multiple sclerosis. Mult Scler.

[8] Marrie RA, Cutter G, Tyry T, Hadjimichael O, Campagnolo D, Vollmer T (2005). Validation of the NARCOMS registry: fatigue assessment. Mult Scler.

[9] Salter AR, Tyry T, Vollmer T, Cutter GR, Marrie RA (2013). “Seeing” in NARCOMS: a look at vision-related quality of life in the NARCOMS registry. Mult Scler.

[10] Motl RW, Schwartz CE, Vollmer T (2009). Continued validation of the Symptom Inventory in multiple sclerosis. J Neurol Sci.

[11] King-Kallimanis BL, Oort FJ, Nolte S, Schwartz CE, Sprangers MA (2011). Using structural equation modeling to detect response shift in performance and health-related quality of life scores of multiple sclerosis patients. Qual Life Res.

[12] Schwartz CE, Snook E, Quaranto B, Benedict RH, Vollmer T (2013). Cognitive reserve and patient-reported outcomes in multiple sclerosis. Mult Scler.

[13] Mokkink L, Knol D, Uitdehaag B (2011). Factor structure of Guy’s neurological disability scale in a sample of Dutch patients with multiple sclerosis. Mult Scler.

[14] Chamot E, Kister I, Cutter GR (2014). Bifactor structure of clinical disability in relapsing multiple sclerosis. Mult Scler Rel Disord.

[15] Cella D, Yount S, Rothrock N, Gershon R, Cook K, Reeve B, Ader D, Fries JF, Bruce B, Rose M, on behalf of the PROMIS Cooperative Group (2007). The Patient-Reported Outcomes Measurement Information System (PROMIS): progress of an NIH Roadmap cooperative group during its first two years. Med Care.

[16] Learmonth YC, Motl RW, Sandroff BM, Pula JH, Cadavid D (2013). Validation of patient determined disease steps (PDDS) scale scores in persons with multiple sclerosis. BMC Neurol.

[17] Muthen B, Kaplan D (1985). A comparison of some methodologies for the factor analysis of non‐normal Likert variables. Br J Math Stat Psychol.

[18] Reise SP, Morizot J, Hays RD (2007). The role of the bifactor model in resolving dimensionality issues in health outcomes measures. Qual Life Res.

[19] Ahmed S, Mayo N, Scott S, Kuspinar A, Schwartz C (2011). Using latent trajectory analysis of residuals to detect response shift in general health among patients with multiple sclerosis article. Qual Life Res.

[20] Hays RD, Morales LS, Reise SP (2000). Item response theory and health outcomes measurement in the 21st century. Med Care.

[21] Bjorner JB, Kosinski M, Ware JE (2003). Calibration of an item pool for assessing the burden of headaches: An application of item response theory to the Headache Impact Test (HIT™). Qual Life Res.

[22] Reeve BB, Hays RD, Bjorner JB, Cook KF, Crane PK, Teresi JA, Thissen D, Revicki DA, Weiss DJ, Hambleton RK, Liu H, Gershon R, Reise SP, Lai JS, Cella D, on behalf of the PROMIS Cooperative Group (2007). Psychometric evaluation and calibration of health-related quality of life item banks: plans for the Patient-Reported Outcomes Measurement Information System (PROMIS). Med Care.

[23] Reise SP (2012). Invited Paper: The rediscovery of bifactor measurement models. Multivariate Behav Res.

[24] Muthén B, Asparouhov T (2013). BSEM measurement invariance analysis. Mplus Web Notes.

[25] Reise SP, Moore TM, Haviland MG (2010). Bifactor models and rotations: Exploring the extent to which multidimensional data yield univocal scale scores. J Pers Assess.

[26] Reise SP, Horan WP, Blanchard JJ (2011). The challenges of fitting an item response theory model to the Social Anhedonia Scale. J Pers Assess.

[27] Andrich D (1988). Rasch Models for Measurement.

[28] Thissen D, Pommerich M, Billeaud K, Williams VS (1995). Item response theory for scores on tests including polytomous items with ordered responses. Applied Psychol Meas.

[29] Orlando M, Sherbourne CD, Thissen D (2000). Summed-score linking using item response theory: Application to depression measurement. Psychol Assess.

[30] Zou G (2004). A modified poisson regression approach to prospective studies with binary data. Am J Epidemiol.

[31] Andrich D (2004). Controversy and the Rasch model: A characteristic of incompatible paradigms?. Med Care.

[32] Hobart JC, Cano SJ, Zajicek JP, Thompson AJ (2007). Rating scales as outcome measures for clinical trials in neurology: problems, solutions, and recommendations. Lancet Neurol.

[33] Edelen MO, Reeve BB (2007). Applying item response theory (IRT) modeling to questionnaire development, evaluation, and refinement. Qual Life Res.

[34] McHorney CA, Monahan PO (2004). Postscript: Applications of Rasch analysis in health care. Med Care.

[35] Reise SP, Bonifay WE, Haviland MG (2013). Scoring and modeling psychological measures in the presence of multidimensionality. J Pers Assess.

[36] Herrmann A, Pfister HR (2013). Simple measures and complex structures: Is it worth employing a more complex model of personality in Big Five inventories?. J Res Pers.

[37] Embretson S, Reise S (2000). Item Response Theory for Psychologists.

