# Supplementary material for: Item response theory-based measure of global disability in multiple sclerosis derived from the Performance Scales and related items
Source: BMC Neurol. 2014 Oct 3;14:192. doi: 10.1186/s12883-014-0192-1 (PMC4195863; doi:10.1186/s12883-014-0192-1)
Supplement: Additional file 1: — Criterion validity of Performance Scale Sum (PSS, raw total score) and individual Performance Scales in cross-sectional studies. [file 12883_2014_192_MOESM1_ESM.docx]

**Additional file 1**

**Criterion validity of Performance Scale Sum (PSS, raw total score) and individual Performance Scales in cross-sectional studies.**

| Performance Scale | Criterion measure | Correlation (absolute value) |
| --- | --- | --- |
| Raw total score (8 item version) | EDSS | 0.62-0.64 ^1, 2^ |
| Raw total score (8 item version) | MSFC | 0.58 ^2^ |
| Mobility | Timed 25-foot walk | 0.77 ^2^ |
| Hand function | Nine hole peg | 0.59 ^2^ |
| Spasticity | No data available | - |
| Bowel/Bladder | Bladder control scale (MSQLI) | 0.75 ^2^ |
| Tremor/Coordination | EDSS | 0.50 ^3^ |
| Tremor/Coordination | Timed 25-foot walk | 0.47 ^3^ |
| Tremor/Coordination | Nine hole peg | 0.51 ^3^ |
| Tremor/Coordination | Cerebellar functional system | 0.51 ^3^ |
| Sensory | Sensory functional system | 0.39 ^2^ |
| Sensory | MOS Pain Effects Scale | 0.64 ^2^ |
| Cognition | PASAT-3 | 0.17 ^2^ |
| Cognition | Mental functional systems | 0.67 ^2^ |
| Cognition (vision) | Perceived deficit questionnaire | 0.71 ^2^ |
| Fatigue | Mod. fatigue impact scale (MSQLI) | 0.76 ^4^ |
| Fatigue | Fatigue Severity Scale | 0.75 ^4^ |
| Pain | Pain Effects Scale (MSQLI) | 0.61-to-0.64 ^5^ |
| Depression | CES-D | 0.73 ^6^ |
| Vision | Low contrast acuity | 0.29 ^7^ |
| Vision | Visual impairment scale (MSQLI) | 0.66 ^7^ |
| Vision | Visual functional questionnaire-25 | 0.70 ^7^ |

PS, original Performance Scales^1^ and assimilated scales^3,5,6^; EDSS, Expanded Disability Status Scale; MSFC, Multiple Sclerosis Functional Composite; MSQLI, Multiple Sclerosis Quality of Life Inventory; PASAT-3, Paced Auditory Serial Addition Test; CES-D Center for Epidemiologic Studies Depression Scale.

Numbers in superscripts corresponds to references below.

Note: The cognition PS, like other patient-reported measures of cognition, correlated more highly with measures of mental distress than with clinical measures of cognitive functioning [2]; The sensory PS is known to exhibit some degree of response shift (patients overestimate their sensory disability early during disease course) [8]. No data are available on the criterion validity of the spasticity PS [2].

**References:**

1. Schwartz CE, Vollmer T, Lee H. **Reliability and validity of two self-report measures of impairment and disability for MS.** [**North American Research Consortium on Multiple Sclerosis Outcomes Study Group.**](http://www.ncbi.nlm.nih.gov/pubmed/9921850) *Neurology* 1999,**52**:63-70.

2. Marrie RA, Goldman M. **Validity of performance scales for disability assessment in multiple sclerosis**. *Mult Scler* 2007,**13**:1176-1182.

3. Marrie RA, Goldman M. **Validation of the NARCOMS Registry: Tremor and Coordination Scale**. *Int J MS Care*. 2011,**13**:114-120.

4. Marrie RA, Cutter G, Tyry T, Hadjimichael O, Campagnolo D, Vollmer T. **Validation of the NARCOMS registry: fatigue assessment**. *Mult Scler* 2005,**11**:583-584.

5. Marrie RA, Cutter G, Tyry T, Hadjimichael O, Vollmer T. **Validation of the NARCOMS Registry: pain assessment**. *Mult Scler* 2005,**11**:338-342.

6. Marrie RA, Cutter G, Tyry T, Campagnolo D, Vollmer T. **Validation of NARCOMS Depression Scale**. *Int J MS Care* 2008,**10**:81-84.

7. Salter AR, Tyry T, Vollmer T, Cutter GR, Marrie RA. **“Seeing” in NARCOMS: a look at vision-related quality of life in the NARCOMS registry**. *Mult Scler* 2013,**19**:953-960.

8. King-Kallimanis BL, Oort FJ, Nolte S, Schwartz CE, Sprangers MA. **Using structural equation modeling to detect response shift in performance and health-related quality of life scores of multiple sclerosis patients**. *Qual Life Res* 2011,**20**:1527-1540.
