# Supplementary material for: Item response theory-based measure of global disability in multiple sclerosis derived from the Performance Scales and related items
Source: BMC Neurol. 2014 Oct 3;14:192. doi: 10.1186/s12883-014-0192-1 (PMC4195863; doi:10.1186/s12883-014-0192-1)
Supplement: Additional file 2: — Methodological details. [file 12883_2014_192_MOESM2_ESM.docx]

**Additional file 2**

**Methodological details**

***1. Exploratory Factor Analysis (EFA)***

We conducted EFA on polychoric correlations in the development sample to obtain preliminary information as to whether the PS should be aggregated into one or more than one disability scales [1,2]. EFA yielded eigenvalues of 5.18, 1.17, and 0.75 for the first three factors. The first factor explained 47% of total variance; the ratio of first to second eigenvalue was equal to 4.44. These results did not clearly indicate whether information contained in the PS were the reflection of one or two underlying factors.

***2. Confirmatory Factor Analysis (CFA)***

We fitted categorical confirmatory factor analysis (CCFA) models [3] to data of the validation sample (i.e., CFA on polychoric correlations among PS using robust weighted least squares with mean and variance adjustment estimation [WLSMV] in MPlus). Comparisons of CCFA models were based on the root mean square error of approximation (RMSEA), the comparative fit index (CFI), and the Tucker Lewis Index (TLI). All three indexes range from 0 to 1. Fit is considered acceptable when RMSEA is ≤ 0.08, CFI ≥ 0.90 and TLI ≥ 0.95 [4]. As supplemental criteria, we also examined the size of standardized factor loadings*,* (≥ 0.30 to be considered salient) and mean absolute residual correlations (≤ 0.10 for good fit) [5].

Results were as follows. *Unidimensional model*: RMSEA, 0.113; CFI, 0.930; TLI, 0.913; mean standardized loadings, 0.65 (range, 0.50-0.77); mean absolute residual correlation, 0.054 (6 values ≥0.10 out of 55). *Two-dimensional model*: RMSEA, 0.093; CFI, 0.954; TLI, 0.941; range of standardized loadings for the PS on their respective factor, 0.52-to-0.80 (mean of 0.69 for the physical disability factor and 0.67 for the mental disability factor); mean absolute residual correlations, 0.039 (range, 0-to-0.143; only 5 values ≥0.01). *Bifactor model*: RMSEA, 0.058; CFI, 0.986; TLI, 0.977; standardized loadings, see article and Figure 2; mean absolute residual correlations, 0.021 (range, 0-to-0.062). Explained common variance (ECV) was 0.73. Percentage of correlations uncontaminated by multidimensionality (PUC) was 0.64. Omega_H_ was 0.79 and Omega_Total_ 0.90. When PUC is <0.80, ECV >0.60 and Omega_H_ >0.70-0.80, the unidimensional solution is preferred over the bifactor solution even if RMSEA, CFI and TLI criteria are not fully met [6].

***3. Revision of PS response options***

Based on results of preliminary analyses conducted in the development sample and replicated in the validation sample, we collapsed a pair of adjacent response options for each PS except the fatigue PS. We collapsed the “Severe” and “Total” disability categories of the vision PS because less than 0.5% of respondents reported suffering from total visual disability. We combined the categories “Frequent use of cane” and “Severe gait disability” of the mobility PS because the probability of selecting the latter category over the former category did not increase as expected as a function of the mean raw score calculated from the other 10 PS (violation of the monotonicity assumption of IRT models). Finally we collapsed the “Severe” and “Total” disability categories of the depression PS, the “Moderate” and “Severe” disability categories of the hand PS, and the “Mild” and Moderate” disability categories of the cognition, bladder/bowel, spasticity, tremor/coordination, cognition, and pain PS, because exploratory IRT analysis indicated that respondents with the same estimated level of perceived disability choose either of these response options with nearly equal probability.

***4. Assessment of measurement equivalence***.

In this study, measurement equivalence (i.e., absence of differential item functioning or DIF) [7]) implied that equally-disabled participants should respond to the PS in the same way regardless of their personal characteristics. Given the time-consuming nature of DIF assessment with IRT method, we used an approach based on multi-group CFA [8,9].

We examined measurement equivalence between groups defined a priori based on gender, ethnicity (African Americans vs. Whites), employment status (employed vs. unemployed), age at assessment (≤ 46 years vs. > 46 years), age at first symptoms (≤ 30 years vs. > 30 years), disease duration (≤ 13 years vs. > 13 years), and DMT at assessment (yes vs. no). Measurement equivalence was assessed at the instrument level to determine if summary scores estimated from all 11 PS could be meaningfully compared across patient groups, and at the item level to determine if individual characteristics influenced how equally-disabled participants responded to each specific PS. Equivalence at the instrument level was assumed if constraining CFA model parameters (loadings and thresholds) to be identical in the compared groups resulted in no more than negligible change in global model fit (CFI difference of less than 0.002 [8]). We assessed measurement equivalence at the item level by estimating between-group differences in factor loadings and thresholds in 1000 bootstrap samples [9]. The hand function PS was selected as referent item because it exhibited stable item functioning in preliminary analysis. We used Benjamini-Hochberg correction for multiple comparisons to determine if a group difference in item loading (respectively thresholds) was statistically significant at the P<0.05 level [10]. We focused attention on PS that displayed DIF corresponding to ≥0.25 effect size in both the development and the validation samples [11].

Results suggested measurement equivalence at the instrument level. At the item level, analyses supported equivalence of all the factor loadings and of threshold parameters for the hand, spasticity, tremor/coordination and pain PS. For the other PS, equivalence of threshold parameters held for some, but not all of the group comparisons. When present, DIF in the thresholds was typically of small-to-moderate magnitude (0.25-to-0.40 effect size) except at the extreme ends of the global disability continuum.

In summary, results of unidimensional CFA suggested that IRT scores estimated from the 11 PS might be meaningfully compared across groups. At the item level, the most salient findings were that, compared to respondents who were younger, employed and/or had a shorter disease duration, *equally-disabled* respondents who were older, unemployed, and/or had a longer disease duration tended to slightly overestimate their mobility and bladder/bowel disability, and underestimate their cognitive, fatigue, sensory, depression and visual disability. These results suggest that **t**he observed pattern of DIF might have resulted from a process of recalibration where the relative importance that persons living with MS attach to the domains of physical and mental disability changes over disease course [12,13].

***5. Main IRT analysis***.

In IRT analysis, we fitted graded response models (GRM) to the data [14]. GRM are appropriate for Likert-type items such as the PS. Basic introductions to IRT and the GRM can be found in [15,16]. Although many fit statistics have been proposed to assess IRT model fit, none is considered entirely trustworthy [6]. When a CCFA model fit the data, the corresponding GRM typically fit the data as well or better [17]. In summary, results of model fit were as follow. *Unidimensional GRM*: M_2_ statistic [18],) 5933.33 for 960 degrees of freedom (P<0.0001); RMSEA, 0.03; Akaike Information Criterion (AIC), 111434.2. *Bifactor GRM*: M_2_ statistic, 3733.67 for 992 degrees of freedom (P<0.0001); RMSEA, 0.03; AIC, 110156.4.

What we referred to in the article as IRT pattern scores were Bayesian Expected a Posteriori scores. IRT summed scores were EAP summed scores [19].

**References**

1. Muthen B, Kaplan D. **A comparison of some methodologies for the factor analysis of non‐normal Likert variables**. *Br J Math Stat Psychol* 1985,**38**:171-189.

2. Reise SP, Morizot J, Hays RD. **The role of the bifactor model in resolving dimensionality issues in health outcomes measures**. *Qual Life Res* 2007,**16**:19-31.

3. Wirth R, Edwards MC. **Item factor analysis: current approaches and future directions**. *Psychol Methods* 2007,**12**:58-79.

4. Hu L, Bentler PM. **Cutoff criteria for fit indexes in covariance structure analysis: Conventional criteria versus new alternatives**. *Struct Equ Modeling* 1999,**6**:1-55.

5. Reeve BB, Hays RD, Bjorner JB, Cook KF, Crane PK, Teresi JA, Thissen D, Revicki DA, Weiss DJ, Hambleton RK, Liu H, Gershon R, Reise SP, Lai JS, Cella D, on behalf of the PROMIS Cooperative Group. **Psychometric evaluation and calibration of health-related quality of life item banks: plans for the Patient-Reported Outcomes Measurement Information System (PROMIS)**. *Med Care* 2007,**45**(Suppl 5):22-31.

6. Reise SP, Scheines R, Widaman KF, Haviland MG. **Multidimensionality and structural coefficient bias in structural equation modeling: A bifactor perspective**. *Educ Psychol Meas* 2013,**73**:5-26.

7. Teresi JA, Fleishman JA. **Differential item functioning and health assessment**. *Qual Life Res* 2007,**16**:33-42.

8. Meade AW, Johnson EC, Braddy PW. **Power and sensitivity of alternative fit indices in tests of measurement invariance**. *J Applied Psychol* 2008,**93**:568-592.

9. Cheung GW, Lau RS. **A direct comparison approach for testing measurement invariance**. *Organ Res Meth* 2012,**15**:167-198.

10. Benjamini Y, Hochberg Y. **Controlling the false discovery rate: a practical and powerful approach to multiple testing**. *J R Stat Soc Ser B* 1995,**57**:289-300.

11. Hambleton RK. **Good practices for identifying differential item functioning**. *Med Care* 2006,**44**(Suppl 3):182-188.

12. Sprangers MA, Schwartz CE. **Integrating response shift into health-related quality of life research: a theoretical model**. *Soc Sci Med* 1999,**48**:1507-1515.

13. Oort FJ. **Using structural equation modeling to detect response shifts and true change**. *Qual Life Res* 2005,**14**:587-98.

14. Samejima F. **Graded response model**. In *Handbook of modern Item Response Theory*. Edited by van der Linden W, Hambleton RK. New York, NY: Springer Verlag; 1997:85-100.

15. Hays RD, Morales LS, Reise SP. **Item response theory and health outcomes measurement in the 21st century**. *Med Care* 2000,38(Suppl 9):28-42.

16. Reise SP, Haviland MG. **Item response theory and the measurement of clinical change**. *J Pers Assess* 2005,**84**:228-238.

17. Maydeu-Olivares A, Cai L, Hernández A. **Comparing the fit of item response theory and factor analysis models**. *Struct Equ Modeling* 2011,**18**:333-356.

18. Maydeu-Olivares A, Joe H. **Limited information goodness-of-fit testing in multidimensional contingency tables**. *Psychometrika* 2006,**71**:713-732.

19. Thissen D, Pommerich M, Billeaud K, Williams VS. **Item response theory for scores on tests including polytomous items with ordered responses**. *Applied Psychol Meas* 1995,**19**:39-49.
