# Supplementary material for: Item response theory-based measure of global disability in multiple sclerosis derived from the Performance Scales and related items
Source: BMC Neurol. 2014 Oct 3;14:192. doi: 10.1186/s12883-014-0192-1 (PMC4195863; doi:10.1186/s12883-014-0192-1)
Supplement: Additional file 3: — Performance Scales Sum (PSS-11) score to IRT summed score of global disability conversion table. [file 12883_2014_192_MOESM3_ESM.docx]

**Additional file 3**

**Performance Scales Sum (PSS-11) score to IRT summed score of global disability conversion table**

| Raw sum score (PSS-11) | IRT score of global disability  Scaled score (M=50, SD=15) | | Standard error  of measurement |
| --- | --- | --- | --- |
| 0 | 8.8 | 8.3 | |
| 1 | 14.4 | 7.4 | |
| 2 | 18.4 | 6.9 | |
| 3 | 21.7 | 6.6 | |
| 4 | 24.6 | 6.4 | |
| 5 | 27.3 | 6.2 | |
| 6 | 29.7 | 6.1 | |
| 7 | 32.0 | 6.0 | |
| 8 | 34.1 | 5.9 | |
| 9 | 36.2 | 5.9 | |
| 10 | 38.1 | 5.8 | |
| 11 | 40.0 | 5.8 | |
| 12 | 41.9 | 5.8 | |
| 13 | 43.7 | 5.8 | |
| 14 | 45.5 | 5.8 | |
| 15 | 47.2 | 5.8 | |
| 16 | 49.0 | 5.8 | |
| 17 | 50.7 | 5.8 | |
| 18 | 52.4 | 5.8 | |
| 19 | 54.1 | 5.8 | |
| 20 | 55.8 | 5.8 | |
| 21 | 57.5 | 5.8 | |
| 22 | 59.3 | 5.8 | |
| 23 | 60.9 | 5.8 | |
| 24 | 62.6 | 5.9 | |
| 25 | 64.3 | 5.9 | |
| 26 | 66.1 | 5.9 | |
| 27 | 67.8 | 5.9 | |
| 28 | 69.6 | 5.9 | |
| 29 | 71.4 | 5.9 | |
| 30 | 73.2 | 5.9 | |
| 31 | 75.1 | 5.9 | |
| 32 | 77.0 | 5.9 | |
| 33 | 78.9 | 5.9 | |
| 34 | 80.8 | 5.9 | |
| 35 | 82.8 | 5.9 | |
| 36 | 84.8 | 6.0 | |
| 37 | 86.9 | 6.0 | |
| 38 | 89.1 | 6.0 | |
| 39 | 91.3 | 6.1 | |
| 40 | 93.7 | 6.2 | |
| 41 | 96.1 | 6.3 | |
| 42 | 98.7 | 6.5 | |
| 43 | 101.5 | 6.6 | |
| 44 | 104.5 | 6.9 | |
| 45 | 107.9 | 7.2 | |
| 46 | 112.0 | 7.8 | |

Example: A patient with a Performance Scales Sum (PSS-11) score of 12 is expected to have a PS IRT score of global disability of 41.9. The standard error of measurement of this score is 5.8. In the sample of NARCOMS registrants used for the development of the PS IRT scale, >99% of pattern scores of global disability range between 5 and 95.

Raw sum score (PSS-11) calculation is based on revised PS response options described in Additional file 2.3.
